# Supplementary material for: Creatine kinase and its muscle/brain isoenzyme as potential biomarkers for cardiovascular disease risk: A cross-sectional analysis of a population-based cohort
Source: PLoS One. 2025 Dec 5;20(12):e0338025. doi: 10.1371/journal.pone.0338025 (PMC12680164; doi:10.1371/journal.pone.0338025)
Supplement: Attachment — Submitted filename: Response to reviewers.docx [file pone.0338025.s001.docx]

**Dear Editor,**

We sincerely appreciate your constructive feedback and the opportunity to revise and resubmit our manuscript. We have carefully considered all editorial and reviewer comments and revised the manuscript accordingly. Below, we provide a detailed point-by-point response to the editorial requirements and comments. Revised sections have also been highlighted in the revised version for your convenience.

We would like to note one minor editorial adjustment made during this revision. To increase accuracy and consistency with the study design, the publication title has been changed to indicate that the analysis is ***cross-sectional*** rather than ***prospective.*** **The study's scope and content remain unchanged.**

**Response to Journal Requirements**

**1. Please ensure that your manuscript meets PLOS ONE's style requirements, including those for file naming.**

***Response:*** We have revised the entire manuscript and supporting files to align with PLOS ONE’s style guidelines and file-naming conventions, as specified in the provided template links. All headings, tables, figures, citations, and document structures now follow the required journal format.

**2. Thank you for stating the following in the Acknowledgments Section of your manuscript:**

**“The authors gratefully acknowledge the financial support (Grant 941822) from the Vice-Chancellor for Research at Mashhad University of Medical Sciences, Iran. The results described in this paper were part of a Pharmacy student thesis.”**

**We note that you have provided funding information that is not currently declared in your Funding Statement. However, funding information should not appear in the Acknowledgments section or other areas of your manuscript. We will only publish funding information present in the Funding Statement section of the online submission form.**

**Please remove any funding-related text from the manuscript and let us know how you would like to update your Funding Statement. Currently, your Funding Statement reads as follows:**

**“The author(s) received no specific funding for this work.”**

**Please include your amended statements within your cover letter; we will change the online submission form on your behalf.**

***Response:*** As requested, we have removed funding-related text from the Acknowledgments section. The revised Funding Statement now reads as follows:

**“**The author(s) received no specific funding for this work.”

The Acknowledgments section has been retained only for non-financial contributions.

**“**We warmly thank the patients who participate in clinical research. Their kindness and trust enable medical progress. We are grateful to everyone who contributed to the MUMS Persian Cohort Study, including the participants. Their diligence and participation were critical to the success of this project. We are grateful to our colleagues and mentors for their crucial support and guidance. Finally, we thank our university for providing an outstanding environment for our studies, which allowed us to complete this pharmacy thesis. ”

**3. In the online submission form you indicate that your data is not available for proprietary reasons and have provided a contact point for accessing this data. Please note that your current contact point is a co-author on this manuscript. According to our Data Policy, the contact point must not be an author on the manuscript and must be an institutional contact, ideally not an individual. Please revise your data statement to a non-author institutional point of contact, such as a data access or ethics committee, and send this to us via return email. Please also include contact information for the third party organization, and please include the full citation of where the data can be found.**

***Response:*** In compliance with your Data Policy, the data availability statement has been revised to indicate a non-author institutional contact point as follows:

**“The datasets generated and analyzed during the current study are not publicly available due to ethical restrictions and privacy concerns regarding human participant data, as mandated by the Institutional Review Board (IRB) of Mashhad University of Medical Sciences. However, de-identified data can be made available to qualified researchers upon reasonable request, subject to approval by the MUMS IRB. Data access inquiries should be directed to the MUMS Ethics Committee at ethics@mums.ac.ir or** [**cohort@mums.ac.ir**](mailto:cohort@mums.ac.ir)**.”**

**4. Your ethics statement should only appear in the Methods section of your manuscript. If your ethics statement is written in any section besides the Methods, please delete it from any other section.**

***Response:*** The ethics statement has been retained solely within the Materials and Methods section. All duplicate mentions in other have been removed.

**Response to Additional Editor Comments**

1- **I congratulate the esteemed authors for their unique approach to this important topic. Your manuscript is generally well written, but there are some issues that need to be addressed.**

***Response:*** We thank Editor for their supportive comments highlighting the value of our study.

2- **The introduction and materials and methods sections are too long. You could shorten the paragraphs in the introduction that describe the biochemical metabolic functions of creatine phosphokinase or move them to the discussion section, avoiding repetition, of course.**

***Response:*** We have carefully shortened the Introduction by removing redundant biochemical explanations of creatine phosphokinase. Relevant mechanistic details were relocated to the Discussion section where appropriate.

The Materials and Methods section was also condensed for clarity and reduced repetition.

3- **In the methodology section, the “Sample Collection and Laboratory Procedures” section is repeated twice; please combine these.**

***Response:*** The duplicated subsections have been merged into a single, streamlined section entitled “Study design and participants” to eliminate redundancy.

4- **In the “Sample Size Considerations” section, you state that you included 246 cases from 7,000 applications that met the study criteria. It would be helpful to add a flow chart on this topic and explain in the discussion section why you were able to obtain such a low number.**

***Response:*** Thank you for your thoughtful and constructive feedback on our manuscript. You raise an excellent point regarding the need for greater clarity on our sample selection process. We agree that the journey from 7,000 applications to the final 246 analyzed cases is a critical aspect of our methodology that warrants a more detailed explanation. We have added a new paragraph to the Discussion section (also detailed below) that explicitly addresses the factors leading to the final sample size. We explain why this number, while a subset of the total pool, is both expected in this research context and robust for the statistical analyses performed.

***“*A comment about the sample size is necessary. Out of the initial 7,000 cohort applicants, 246 satisfied the tight inclusion criteria and were selected for analysis. This reduction is attributable not to inadequate participation, but to the intentional elimination of incomplete records and those without essential demographic, biochemical, or cardiovascular information. The multi-stage screening yielded a uniform, well-defined sample while reducing extraneous variables. Although smaller than the original pool, this analytical selection demonstrated sufficient statistical power for the exploratory investigations performed."**

5- **I believe that not specifying the gender numbers in Table 1 is a major omission.**

***Response:*** We appreciate the editor's suggestion. Because Table 1 only shows continuous variables (mean ± SD, min, max), it doesn't include categorical data like sex. But we have explicitly said how many men and women took part and what percentage of them were men and women in the Results section (3.1): "Of the 246 people who took part, 103 (41.9%) were men and 143 (58.1%) were women."

6- **Please add a section to the discussion section before the conclusion heading that explains the strengths and limitations of your study.**

***Response:*** A new subsection titled Strengths and Limitations has been inserted immediately before the Conclusion. This section acknowledges study constraints (e.g., limited sample representation) and highlights the strengths, such as rigorous diagnostic criteria and standardized laboratory procedures.

**Response to Reviewer Comments**

**Reviewer #1**

1. **The introduction part is over reaching in it’s relation between ck and ckmb and electrolyte imbalance .**

***Response:*** We appreciate this observation. The introduction has been revised to focus more directly on the established physiological role of CK and CK-MB in cardiovascular health. The extended mechanistic discussion of electrolyte imbalance was condensed, with only the most relevant references retained to maintain focus and balance.

2. **Rigorous methodology but excessive details. Needs trimming.**

***Response:*** The methodology section has been shortened by removing redundancies (e.g., duplicate descriptions of sample collection) and summarizing technical details, while still retaining sufficient information to ensure reproducibility.

3. **Due to the limited sample size, the focus was on descriptive analysis and assessing associations rather than hypothesis testing.**

**shouldn’t the main study primary and secondary goals have been already established in the protocol at clinicaltrails.gov does this mean they were changed.. This point needs clarification.**

***Response:*** Thank you for raising this point. The present study was designed as a descriptive and observational analysis using data derived from the MUMS Persian Cohort, rather than as a hypothesis-driven clinical trial. Accordingly, no formal sample size calculation was performed, and the study was not pre-registered in ClinicalTrials.gov, as it did not involve any interventional procedures or prospective outcome testing.

The primary objective of this work was exploratory to describe the relationships between serum CK/CK-MB levels and cardiovascular risk factors and to generate hypotheses for future confirmatory studies.

4. **individuals with low muscle mass are excluded but it doesn’t say the cut off BMI for exclusion and it doesn’t add the BMI in the descriptive statistics section or comment on it in the manuscript statistics.**

***Response****:* We agree this was unclear. We have now specified that individuals with **BMI < 18.5 kg/m²** were excluded as proxy for low muscle mass. BMI has been added to the descriptive statistics table, and its relevance is acknowledged in the results and discussion.

**5. Patients that use of captopril, colchicine, alcohol, lovastatin, lithium, lidocaine, and propranolol.**

**This need explanation to why patients using these specific drugs specifically were excluded and why other categories of drugs were not excluded like ccb and diuretics ( for example.. Why captopril specifically and not whole ACEI class).**

**•Also since some of the sample size were hypertensive patients on treatment wouldn’t some of the will be on diuretics that can attribute to some form of electrolytes imbalance or renal impairment . This needs to be addressed (at least in the limitation section)**

***Response:*** We thank the reviewer for this important comment. These specific drugs were excluded because they are known to **directly alter CK/CK-MB levels** (e.g., statins, colchicine, alcohol) or interfere with electrolyte balance and muscle metabolism. We agree that the rationale was not sufficiently explained. The revised manuscript now explicitly justifies these exclusions and acknowledges in the limitations section that we did not exclude all possible drug classes (e.g., calcium channel blockers, diuretics). We now discuss the possible confounding influence of diuretic therapy in hypertensive patients.

**6. Across the whole study population, regardless of gender, a significant poor correlation (r < 0.29) was found between log CK and potassium, creatinine, systolic blood pressure (SBP), and diastolic blood pressure (DBP).**

**A significant effect of log CK on DBP and CVH was found , meaning that each associated variable’s value rose with every unit increase in log CK.**

**(The first sentence contradicts the second sentence regarding DBP please elaborate on this more)**

***Response:*** We thank the reviewer for this astute observation. The apparent contradiction arises from the distinction between a simple bivariate correlation and a multivariable regression model.

The poor correlation (r < 0.29) describes the isolated, linear relationship between log CK and DBP, which is indeed weak when considered alone. However, the significant effect in the regression model indicates that after accounting for the influence of other variables in the model (e.g., age, BMI, medications), log CK exerts a small but statistically independent effect on DBP. We have clarified this distinction in the revised manuscript.

**7. The results section can be rewritten for clarity (to be less confusing).**

***Response:*** The results have been rewritten to improve flow and avoid confusion. Tables and text now consistently align with one another.

**8. “Log CK is a significant predictor of gender—is it muscle mass instead?”.**

***Response:*** We appreciate this insightful comment. We agree that the phrasing “log CK is a significant predictor of gender is potentially misleading for a cross-sectional, observational analysis. In biomarker epidemiology, higher CK in men is widely attributed to greater skeletal muscle mass, not to sex per se as a causal factor. We have therefore

replaced “gender” with “sex (male vs. female)” throughout; reworded the Results to state that log CK differed by sex, and that this difference is likely explained by muscle mass differences; clarified in *Statistical Analysis* that we treat sex as a covariate, and we note BMI as a pragmatic surrogate for muscle mass in sensitivity considerations; added a statement in the *Discussion* acknowledging that we did not directly measure muscle mass; thus sex differences in CK should be interpreted as muscle-mass–related rather than as sex being “predicted” by CK.

**9. log CK levels emerged as significant predictors of CVH but Log CK-MB was found to have a significant poor correlation (r < 0.29) with both SBP and CVH in males ( please try to give an explanation or a comment on that in the discussion).**

***Response:*** We thank the reviewer for this thoughtful point. In brief, total CK reflects systemic energy metabolism and is strongly influenced by skeletal muscle mass, whereas CK-MB is more cardiac-specific and tends to index current/subclinical myocyte stress rather than cumulative history. In our cross-sectional sample, log CK showed a small but statistically significant association with CVH, consistent with a long-term hemodynamic/ion-transport milieu, while in men the log CK-MB correlations with SBP and CVH were weak—plausibly due to CK-MB’s narrow range in non-acute settings, treatment-related SBP range restriction, smaller male subgroup size (reduced precision), and the differing biology that CK-MB reflects ongoing myocardial workload more than past events. We have added a concise clarification to the Discussion and explicitly note that these effects are modest, exploratory, and non-causal, acknowledging limitations including cross-sectional design, potential confounding by muscle mass, and the absence of direct muscle-mass measures.

**10. The discussion section about the hypertension and CK and CKMB is very informative**

**And the acknowledgement of the small sample size is on point.**

***Response:*** We thank the reviewer for the encouraging feedback. We are pleased that the discussion of hypertension in relation to CK and CK-MB was found informative, and we appreciate the recognition of our transparent acknowledgment of the modest sample size.

Considering this comment, we made two small refinements: (i) we tightened the section on generalizability to emphasize that effect sizes should be interpreted cautiously and primarily as hypothesis-generating, and (ii) we added a brief sentence in the Limitations outlining our plan for validation in a larger, longitudinal cohort with direct measures of muscle mass. We trust these minor clarifications further align the manuscript with the reviewer’s expectations.

**11. The manuscript lacks a didicated limitations section.**

***Response****:* We agree and have added a dedicated Limitations section. It merges and expands points previously noted in the Discussion and clarifies constraints related to design, measurement, generalizability, and analysis choices. The new section appears immediately before the conclusion.

**12. The statistics needs to clarify the numbers of males and females and make specific tables for their results and characteristics especially when part of the point of the study is the difference between them, same goes for the number of hypertensive patients.**

***Response:*** We agree that explicit stratification improves clarity. We now state the numbers of males and females and normotensive vs. treated-hypertensive participants in Results 3.1 and reference Table 2 where these counts are already displayed.

**13.** **The study didn't report on the LDL in the lipid profile in the results and the tables.**

***Response:*** We appreciate the reviewer’s comment. LDL-cholesterol (LDL-C) was not systematically available in the cohort dataset for the study period; to avoid biased or incomplete analyses, we pre-specified the Atherogenic Index of Plasma (AIP = log_10_[TG/HDL-C]) as our primary lipid metric. AIP provides a compact indicator of the TG–HDL balance and has been linked to atherogenic lipoprotein patterns, making it suitable for this exploratory, population-based analysis. We have clarified this explicitly in the Methods (Data Collection and Statistical Analysis) and noted in Results that lipid risk is summarized by AIP.

**14. The cardiovascular disease history that was mentioned repeatedly in the study and it's relation to log CK and log CKMB wasn't clearly defined in the results or the tables.**

***Response:*** We appreciate this comment. We now standardize cardiovascular history to “CVH” across the manuscript, provide an operational definition (prior physician-diagnosed myocardial infarction, angina, or stroke; binary 0/1), report its prevalence in the Results, and flag CVH explicitly in tables/legends. We also clarify in the Statistics section that bivariate associations with CVH use point-biserial Pearson correlations, while model summaries treat CVH as a binary outcome in an exploratory linear framework. These updates make the construct and its relation to log CK and log CK-MB unambiguous.

**15. The manuscript needs correlates the results of of log ck and log ckmb to BMI and muscle mass.**

***Response:*** We agree. Because we did not have direct measures of muscle mass, we used BMI as a practical surrogate. We now report log CK–BMI and log CK-MB–BMI associations (overall and by sex) in Results 3.4–3.6 and added BMI rows to the correlation/regression tables. As expected for this exploratory study, the observed effects are small (weak) and should be interpreted cautiously. The Methods now state that BMI is used to contextualize muscle-mass variation, and the Limitations note the absence of direct muscle-mass measurements.

**16. There is a need in the manuscript to elaborate the cause of using the log of ck and ckmb instead of comparing them directly.**

***Response:*** We agree the rationale should be stated clearly. CK and CK-MB are highly right-skewed in community samples, so we log-transformed them in advance to (1) reduce skew and stabilize variance, (2) make relationships with covariates more linear, and (3) reduce the impact of outliers, which helps meet assumptions for Pearson correlations and linear models. For readability, we still report descriptive values in original units, and note that effects on the log scale reflect proportional (percent) differences in CK/CK-MB. These issues are addressed in the revised manuscript.

**17. The correlation of almost all the variables is weak but the manuscript keeps making strong conclusions or at least strong recommendations from this weak correlations.**

***Response:*** We agree and have revised the manuscript to ensure that weak correlations are not overinterpreted. We now consistently frame findings as associations of small magnitude in an exploratory, cross-sectional study. Language implying prediction, causality, or clinical recommendation has been removed or softened. The Abstract, Results, Discussion, and Conclusions were updated to emphasize effect size over statistical significance, and we added explicit caution about multiple comparisons and non-causal inference. These changes align the interpretation with the observed data.

**18. The talk about failure of antihypertensive treatments is not supported by the weak correlation.**

***Response:*** We appreciate this comment and agree that our wording overstated the implications. The study was not designed to assess antihypertensive treatment failure, and observed associations were weak. We have removed language implying treatment failure and now describe hypertension simply as treated vs. normotensive at the time of measurement. We also clarify that therapy may compress blood-pressure variability, which could attenuate cross-sectional associations, but our data do not evaluate treatment efficacy.

**19. All the results of log ckmb are within normal and only some of the results of log ck (not specified in the study how much of them) are abnormal**

**Which negates most of the point of the study (or at least decrease the point of it).**
***Response:*** We appreciate this point. As a population-based, cross-sectional study, we expected most CK-MB values—and many CK values—to fall within clinical reference intervals. Our aim was not diagnostic classification but to examine whether within-range variation in CK and CK-MB is associated (albeit weakly) with cardiovascular measures. To make this explicit, we now (i) define the laboratory reference intervals used for CK and CK-MB, and (ii) state clearly that analyses treat CK/CK-MB as continuous variables, so findings reflect subtle, within-normal-range differences rather than abnormality per se.

**20. Almost all the multivariate results shows no correlation and the paper keeps sourcing the results of the univariate results in the results and discussion section.**

***Response:*** We appreciate this point. In the revised manuscript, we clarify that multivariable (adjusted) results form the basis of inference, and univariate findings are presented only as exploratory context. We added a brief statement in Methods to specify this hierarchy, a one-sentence reminder in the Results sections that adjusted estimates are primary (and that most univariate signals attenuate after adjustment), and a short Discussion sentence reinforcing cautious interpretation. Table legends for univariate summaries now note they are exploratory.

**21. The manuscript talks about the relation between ck and hypertension (ck in other diseases) but doesn't talk about ckmb (in general it should expansion in talking about the relation between ck and ckmb and their relation to other diseases.**

***Response:*** We agree that CK and CK-MB have broader clinical contexts. Given our exploratory, population-based scope, we added a brief bridge in the Discussion noting that CK is largely muscle-derived, CK-MB is relatively cardiac-specific, and that our findings reflect subtle within-range variation, not overt disease states. We keep detailed disease discussions out of scope to maintain analytic focus.

**22. After all the talk of the relation between ck and antihypertensive failure the study doesn't apply this.**

***Response:*** We appreciate this point. Our study was not designed to evaluate antihypertensive treatment failure; analyses were cross-sectional and compared treated-hypertensive with normotensive participants at a single time point. We have removed language implying “treatment failure” and now describe findings strictly as associations observed under treatment. We added brief clarifications in the Abstract, Results (§3.3), and Discussion, and we note this constraint explicitly in the Limitations.

**23. Conclusion overstated.**

***Response:*** The conclusion has been tempered to reflect the exploratory nature of the study and the small effect sizes observed.

**Reviewer #2**

1. **An interesting manuscript regarding cpk and ck mb values and its correlation with gender related factors and other cardiovascular risk factors. Those data are partially known nut the multivariate analysis performed by the authors confirmed this knowledge, establishing very well the correlation between these widely available laboratory parameters and cv risk factors.**

***Response:*** We thank Reviewer #2 for their supportive comments highlighting the value of our study. We are pleased that the reviewer recognized the contribution of our multivariable analysis in confirming and extending existing knowledge on CK and CK-MB as widely available biomarkers related to cardiovascular risk.
